# Translation and validation of the German version of the FACE-Q paralysis module in adult patients with unilateral peripheral facial palsy

**DOI:** 10.1038/s41598-024-58159-8

**Published:** 2024-03-31

**Authors:** Wieta Elin Moritz, Gerd Fabian Volk, Helene Kreysa, Orlando Guntinas-Lichius

**Affiliations:** 1 Department of Otorhinolaryngology, Jena University Hospital, Am Klinikum 1, 07747 Jena, Germany; 2 Facial-Nerve-Center, Jena University Hospital, Jena, Germany; 3 Center for Rare Diseases, Jena University Hospital, Jena, Germany; 4 Department for General Psychology and Cognitive Neuroscience, Friedrich Schiller University, Jena, Germany

**Keywords:** Patient reported outcome measure, Quality of life, Questionnaire, Appearance, Craniofacial, Paralysis, FDI, FaCE, Health care, Signs and symptoms

## Abstract

The aim was to develop and validate a German version of the FACE-Q paralysis module, a patient-reported outcome measure to assess health-related quality of life in adult patients with unilateral facial palsy. The FACE-Q craniofacial questionnaire, which includes the paralysis module, was translated. 213 patients with facial palsy completed the German FACE-Q paralysis along with the established FDI and FaCE questionnaires. Regression analyses were performed to examine the relationships between the different FACE-Q domains and patient and therapy characteristics. The FACE-Q scales had high internal consistency (Cronbach’s alpha all > 0.6). High correlations were found between the FACE-Q and the FDI and FaCE (mean rho = 0.5), as well as within the FACE-Q (mean rho = 0.522). Unifactorial influences were found for all domains except *Breathing* (all p < 0.05). Multivariate independent predictors were found for some FACE-Q domains. Most influential predictors (> 8 subdomains): Patients who received physical therapy scored lower in ten subdomains than those who did not (all p < 0.05). Patients who had surgery scored lower in nine subdomains than patients without surgery (all p < 0.05). The German version of the FACE-Q Paralysis Module can now be used as a patient-reported outcome instrument in adult patients with facial nerve palsy.

## Introduction

The face is arguably the most important part of the human body, playing a significant role in determining an individual’s attractiveness^1^. Facial deformities or disfigurements can have severe psychological consequences^2^. Especially individuals affected by facial nerve palsy can suffer greatly from such facial deformities. These patients may experience impairments across various domains of their quality of life, including diminished self-esteem, psychological and social challenges, as well as difficulties in performing daily activities^3–5^. In order to fully assess these impairments clinically, it is crucial to not only to rely on objective measurement methods but also to consider the subjective perception of patients, particularly their self-perception of their appearance. Since the development and validation of disease-specific patient-reported outcome measures (PROMs), significant progress has been made, and these measures have been implemented in many clinical settings in the field of otorhinolaryngology^6^. For patients with facial palsy, the questionnaires best validated for this purpose to date, the facial disability index (FDI) and the facial clinimetric evaluation (FaCE) scale, have been widely used in clinical routines for several years^5^. A limitation of both questionnaires is the assessment of self-perception of appearance. Since it has been shown that patients with facial palsy may have a higher risk of developing body dysmorphic disorder, it is even more important to capture the appearance from the patient’s point of view^7^. In this context, the patient-reported outcome instrument FACE-Q Paralysis questionnaire, which was developed by Klassen et al. in 2020, stands out as a comprehensive tool for capturing the patient’s perspective in the areas of Appearance, Facial Function, Health-related Quality of Life, and Adverse Effect^8,9^, The FACE-Q Paralysis is part of the more comprehensive FACE-Q Craniofacial questionnaire, which is an outcome measure designed for patients with visible and/or functional facial distinction^9^. The gap of knowledge of the already established questionnaires FDI and FaCE is the assessment of the appearance from the patient’s point of view. This is the reason why this study attempts to fill the gap also for use in clinical settings for German speaking patients. Its focus on the Appearance domain (five out of 16 subdomains) makes it a valuable addition to the existing validated PROMs for patients with facial palsy. The original version of the questionnaire is available in English and has been translated into multiple languages, but not yet into German^8,10^.

In the present study, our goal was therefore to validate a German version of the FACE-Q Craniofacial, of which the FACE-Q Paralysis forms one part, among patients with facial palsy, by comparison with the FDI and FaCE questionnaire. In addition, the aim was to investigate possible independent predictors that could influence the response to the FACE-Q Paralysis questionnaire.

## Materials and methods

This prospective observational study was performed at the Department of Otorhinolaryngology, Jena University Hospital, Jena, Germany. Approval for the study was obtained through the local institutional ethics review board, the Ethics committee of the Friedrich-Schiller-University, Jena, Germany (No. 2022-2695-Bef). Written informed consent was obtained from all study participants, and/or their legal guardians/caregivers. All experimental procedures with human subjects followed the institutional research committee’s ethical standards and the 1964 Helsinki Declaration and its later amendments.

### Translation of the FACE-Q craniofacial module and of the FACE-Q paralysis module

The FACE-Q Craniofacial questionnaire is a PROM instrument intended for patients with visible and or functional facial differences between the ages of 8 and 29 years. The questionnaire consists of four domains representing appearance, function, health-related quality of life (HRQOL) and adverse effects. Each domain is composed of several subdomains, which consist of multiple independently functioning scales^9,11^. While the Craniofacial module is designed to address facial differences overall, the FACE-Q Paralysis module, which is part of the larger FACE-Q Craniofacial, specifically focuses on patients with facial paralysis and has no age limitation. The FACE-Q Paralysis questionnaire consists of the same four domains as the FACE-Q Craniofacial, each with several subdomains. These domains include *Appearance* (includes subdomains *Eyes, Face, Forehead, Lips, Smile*), *Facial Function* (includes subdomains *Breathing, Eating/Drinking, Eyes, Face, Speech*), *Health-related quality of life* (includes subdomains *Appearance Distress, Psychological, Social, Speech Distress*) and *Adverse Effects* (includes subdomains *Eyes, Face*). The questionnaire thus includes 16 subdomains made up a total of 146 questions. Multiple items are included in each FACE-Q scale that can be rated on a 3- to 4-point Likert scale. Depending on the question of the subdomain there were various possible answers (e.g. Not at all, a little bit, quite a bit, very much) between which the patient could choose on the Likert scale. The raw scores of each scale are converted into a range from 0 (worst) to 100 (best) based on the findings of Rasch analysis^12^. Exceptions are the subdomains *Eye Function*, *Eye Adverse Effects* and *Face Adverse Effects,* which are checklists for identifying problems experienced by the patients. These checklists cannot be converted based on Rasch analysis because the sets of items may not function together statistically^8,10^.

For this study, the entire FACE-Q Craniofacial was translated first, but validation in German was done for the FACE-Q Paralysis. A German version of the FACE-Q Craniofacial questionnaire was produced out of the original English version. The translation process and cross-cultural adaption was done following the International Society for Pharmacoeconomics and Outcomes Research (ISPOR) guidelines^13^ as requested by the Q-Portfolio team. Two separate forward translations were performed by native German speakers who were fluent in English. Based on their translation, a reconciled version was agreed on. A backwards translation was done by a native English speaker. The original English version and the backwards translation of the questionnaire were then compared by the Q-Portfolio Team at McMaster University (Hamilton, Canada) and The Hospital for Sick Children (Toronto, Canada), publisher of the original questionnaire. A pilot study was performed to test the comprehensibility of the German version. Six patients, who were fluent in German, were recruited from the outpatient clinic of the Facial-Nerve-Center, Jena University Jena, Germany. After completing the questionnaire, these patients were interviewed to identify any potential difficulties in comprehension and gather suggestions for improving the translation. Any misunderstandings that these patients raised, such as single terms or filler words to improve language comprehension, were improved before finalizing the questionnaire for use in this study. Each of the six patients was able to complete the questionnaire and answer the associated comprehension questions. The German version of questionnaire can now be requested via the website of the Q-Portfolio Team (https://qportfolio.org/face-q/paralysis/).

### The other PROMs: facial disability index (FDI) and facial clinimetric evaluation (FaCE)

In addition to the FACE-Q Paralysis, the two other questionnaires of the survey, the facial disability index (FDI) and the facial clinimetric evaluation (FaCE), have already been validated in German^14^. The FDI consists of 10 questions with Likert-scale response options, subdivided into two parts: *Physical Function* and *Social/Well-being Function*. The *Physical Function* scale ranges from − 25 (worst) to 100 (best), while the *Social/Well-being Function* scale ranges from 0 (worst) to 100 (best)^15^. The FaCE consists of 15 questions with 5-point-Likert scale responses, subdivided into six domains: *Facial Movement, Facial Comfort, Oral Function, Eye Comfort, Lacrimal Control,* and *Social Function*. Each scale ranges from 0 (worst) to 100 (best) and a total score is obtained^16^.

### Patient selection and survey

Selection criteria for the study were fluent German-speaking patients over 8 years of age presenting to the Facial-Nerve-Center in Jena between 2018 and 2022 who were diagnosed with facial nerve disorders. Individuals with both acute and chronic facial nerve palsy were included. Furthermore, the study also invited patients who had already recovered from their facial palsy, thus representing individuals with milder symptoms. The survey consisted of 22 pages, including a one-page cover letter to the patients, one page consisting of seven questions on personal data and the three questionnaires FACE-Q Paralysis, FDI and FaCE (total of 20 pages). A total of 800 patients were contacted by mail between November 2022 and February 2023, of whom 214 patients (response rate: 27%) participated in the survey and returned at least one of the paper-based questionnaires by mail. Inclusion criteria were defined as follows: patient age at least 8 years and they were required to complete at least one of the three questionnaires. For the FDI and FaCE questionnaires, full completion was mandatory, while for the FACE-Q questionnaire a minimum of 50% completion for each subdomain was necessary. The remaining responses for the missing items were derived from the answer that was given most common response for the domain, following the provided instructions for use^9^. One patient had to be excluded because he was under the age of 8 years. By reading the instructions and the information on the data protection policy, and by completing the questionnaires, each patient has given their consent to the collection and processing of their data”.

### Statistical analyses

All statistical calculations were performed with IBM SPSS Statistics (Version 29.0; IBM Corp., USA). Unless otherwise stated, descriptive statistics data are presented in mean, standard deviation (SD), median, range, and relative data in percentages. In order to assess the internal consistency of the questions within the domains of the German version of the FACE-Q, Cronbach’s alpha coefficient was calculated and the 95% confidence interval is provided. Generally, a Cronbach’s alpha coefficient value above 0.7 is considered to indicate acceptable internal consistency^17^. Spearman’s rank correlation coefficients (Spearman’s Rho) were calculated to assess the correlations between items within the FACE-Q and against FDI and FaCE. It is commonly accepted that a correlation coefficient of 0.30–0.59 represents a fair correlation, 0.60–0.79 represents a moderate correlation and values exceeding 0.8 indicate a very strong correlation^18^. Nominal p-values for two-sided testing were used, with a significance level set at p < 0.05. In order to determine which clinical parameters exhibited a statistically significant impact (p < 0.05) on the FACE-Q results, a univariate analysis was performed using the non-parametric Mann–Whitney U Test. This involved dichotomizing the clinical parameters, such as dividing age into two groups above and below the respective median. For multiple significant results in the univariate analyses, the corresponding parameters were subsequently tested for their influence using multiple binary regression analysis. In each case, the regression coefficient B, 95% confidence interval, standard error and significance p are presented. Subdomains that had no significant effect in the univariate analyses and those that were significant only for the two parameters physical therapy and surgery were not considered in the further multivariate analyses. Normality tests were performed for all scales of each questionnaire. Skewness, kurtosis and test values for Shapiro–Wilk test were reported. Maximum likelihood factor analysis was performed on all 16 items of the FACE-Q questionnaire. Kaiser–Meyer–Olkin value (KMO > 0.5) and Bartlett’s test of sphericity (p < 0.05) were performed to show the appropriateness of the values for factor analysis^19^. The number of factors was determined and presented as rotated factor matrix.

## Results

### Patients’ characteristics

Table 1 presents the characteristics of the 213 included patients. The median age of the participants was 57 years. More female than male patients were included (61.5%). The most frequent etiology was an idiopathic facial palsy (44.6% of patients). The duration between initial diagnosis of facial nerve paresis and survey was 72.0 ± 75.8 months (range 1 to 560 months). Therefore, acute and chronic palsy were represented. Over half of the respondents had received physical therapy at some point (59.2%) and a similar proportion had participated in a facial palsy training (58.2%).

**Table 1:** Characteristics for the participants with facial nerve palsy.

	N	%
All	213	100
Gender		
Female	131	61.5
Male	82	38.5
Classification		
Peripheral	133	62.4
Unknown	70	32.9
Central	8	3.8
Nuclear	2	0.9
Etiology		
Idiopathic	95	44.6
Inflammatory	47	22.1
Neoplastic	31	14.6
Iatrogenic postoperative	25	11.7
Traumatic	7	3.3
Congenital	2	0.9
Other	6	2.8
Therapy		
Physical therapy	126	59.2
Facial mimic training	124	58.2
Medication	111	52.1
Surgery	61	28.6
Other	23	10.8

	Mean ± SD	Median; range
Age in years (N = 198*)	55.2 ± 16.4	57.0; 12–86
Interval between onset of palsy and survey in months (N = 181*)	72.0 ± 75.8	55.0; 1–560

*N* sample size, *SD* standard deviation.

*Not all participants answered the respective question.

### Questionnaires: FACE-Q, FDI and FaCE

**Figure 1:**
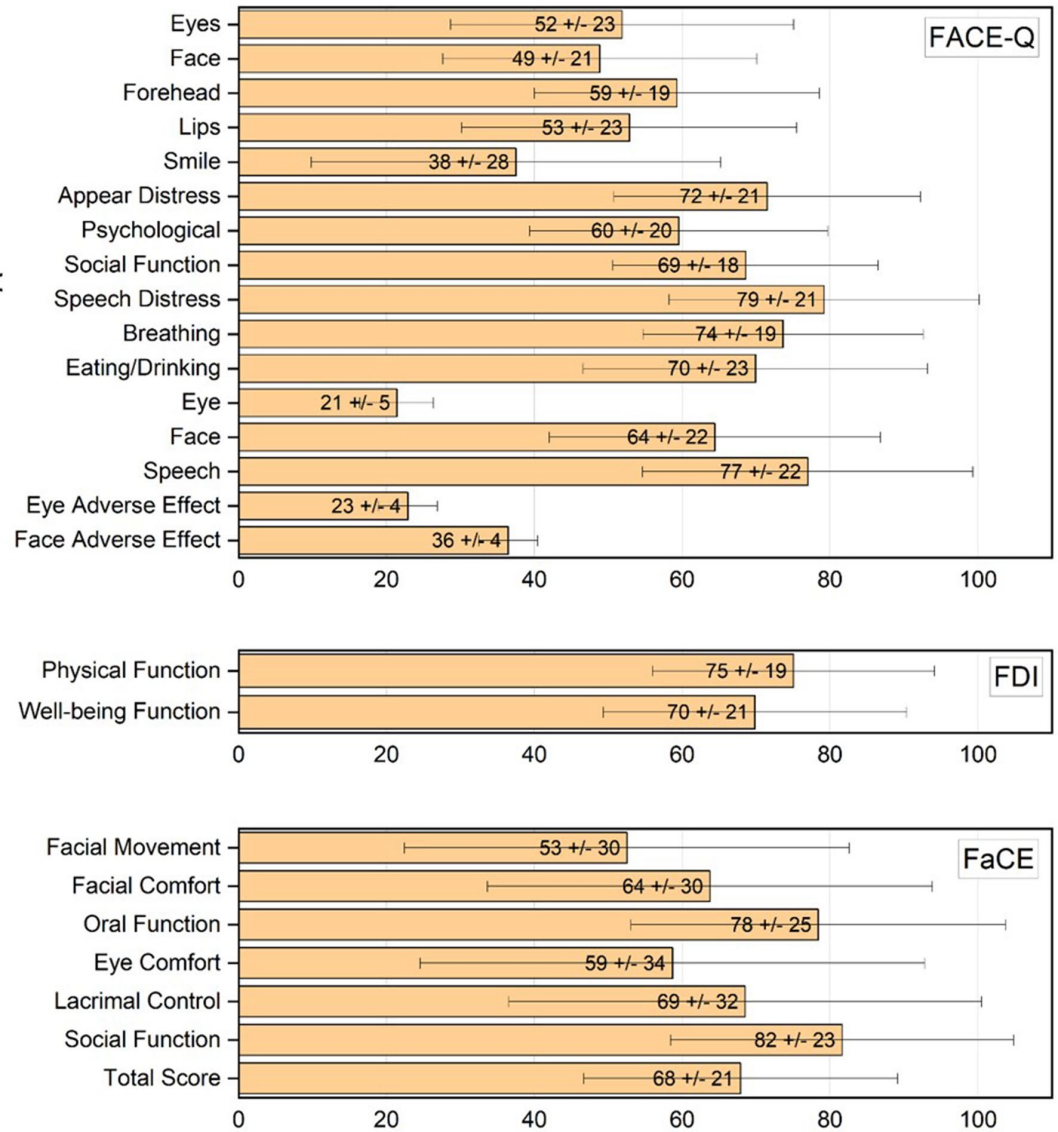
Results of the FACE-Q, FDI and FaCE questionnaires. All domains are presented with mean values and standard deviation.

The results of the FACE-Q, as well as the FDI and FaCE questionnaires, are presented in Fig. 1. The lowest mean FACE-Q subdomain score was observed for *Appearance Smile* with a mean score of 35.7 ± 27.7, while the lowest mean score within the three checklists was found for *Eye Function* (21.4 ± 5.1). For the remaining subdomains *Eyes, Face, Forehead* and *Lips* of the domain *Appearance*, mean scores between 48.9 ± 21.2 and 59.3 ± 19.3 could be determined. It can be observed that, on average, the respondents reported the highest level of impairment in the domain of *Appearance*. The best results could be reached for the subdomains *Eating/Drinking* (79.9 ± 23.3) and *Speech Distress* (79.2 ± 21.0) associated with the domain *Facial Function*. For FDI, a lower mean score was found for the domain *Social/Well-being Function* (69.8 ± 20.5) compared to the domain *Physical Function* (75.0 ± 19.1). The lowest mean score for FaCE was found for the domain *Facial Movement* (52.2 ± 30.1), while the highest score was found for the domain *Social Function* (81.6 ± 23.2).

### Internal consistency of the three questionnaires

The internal consistency of the three questionnaires is shown in Supplementary Table 1. Cronbach’s alpha values for FACE-Q were ≥ 0.771 for 12 scales. Only the subdomain *Breathing* showed a lower Cronbach’s alpha of 0.609. The internal consistency of the FDI and FaCE questionnaires both showed high Cronbach’s alpha values of ≥ 0.759 for FaCE and ≥ 0.791 for FDI. Therefore, the internal consistencies for both questionnaires are similar to those shown in the original German validation of the FDI (≥ 0.835) and FaCE (≥ 0.667)^14^.

### Correlation between the three questionnaires and within the FACE-Q

Supplementary Table 2 shows the correlation between FACE-Q, FDI, and FaCE. The correlation from FACE-Q to both questionnaires varied greatly. For FDI, correlations ranged from rho = 0.316 to rho = 0.758. Best correlations of the domain *Physical Function* existed with rho > 0.630 to the FACE-Q subdomains *Eating/Drinking* (rho = 0.758; p < 0.001) and *Facial Function* (rho = 0.743; p < 0.001). In the domain *Social Function*, the best correlation existed to the FACE-Q subdomain *Social Function* (rho = 0.655; p < 0.001). For FaCE, correlations ranged from rho = 0.203 and rho = 0.828. The domains *Total Score, Oral Function* and *Facial Movement* showed the best correlation to FACE-Q. These domains correlated to the FACE-Q subdomains *Facial Function* (rho = 0.828; p < 0.001). *Eating/Drinking* (rho = 0.787; p < 0.001) and *Facial Function* (rho = 0.786; p < 0.001), respectively. On average, correlations from FACE-Q to FaCE (rho = 0.501) were slightly better compared to FDI (rho = 0.495; except for one correlation, all p < 0.001). Correlations within the questionnaire FACE-Q are shown in Supplementary Tables 3–5. Correlations ranged from correlation coefficient rho = 0.150 (correlation *Breathing* to *Eyes*) to rho = 0.836 (correlation *Smile* to *Face*). The average for FACE-Q was rho = 0.518 (all p < 0.025).

### Univariate analysis of associations between clinical parameters between FACE-Q subdomains

The results of the univariate analysis indicate that 10 of 12 variables had a statistically significant univariate impact on the outcomes of individual subdomains of the FACE-Q (all p < 0.05; cf. Table 2). Variables that affected any of the subdomains were: sex, age, duration, idiopathic cause, neoplastic cause, postoperative cause, drug treated, participation in facial mimic training, physical therapy and surgery. Participation in physical therapy was significantly associated with most subdomains (all subdomains except *Breathing*). Patients who received physical therapy had lower mean scores (range, 20.1 ± 4.9 to 76.7 ± 21.5), indicating more impairments within these subdomains, than patients who did not (range, 23.3 ± 4.8 to 82.8 ± 19.7). The variables inflammatory cause (yes/no) and any therapy (yes/no), both did not reach a significant level in any subdomain and remained at most marginal (all p > 0.05). The subdomain *Breathing* is the only subdomain that was not associated with any of the variables studied (all p > 0.05).

**Table 2:** Associations between the clinical parameters and FACE-Q outcomes.

		FACE-Q															
		Eyes	Face	Fore-head	Lips	Smile	Distress	Psycho-logical	Social	Speech	Breath-ing	Eating	Eye func-tion	Face func-tion	Speech func-tion	Eye AE	Face AE
Sex																	
	**p**	**0.003**	**0.023**	0.815	**0.020**	0.235	**0.001**	**0.045**	0.370	0.632	0.599	**0.013**	0.369	0.152	0.138	0.066	0.058
Male	Mean ± SD	56.5 ± 20.7	51.2 ± 19.5	58.9 ± 19.5	56.5 ± 21.5	49.3 ± 27.0	77.1 ± 19.6	62.1 ± 21.2	69.6 ± 18.7	80.4 ± 20.0	73.0 ± 18.3	75.0 ± 21.6	21.8 ± 4.9	66.8 ± 22.0	74.7 ± 21.6	23.6 ± 3.2	37.0 ± 3.8
Female	Mean ± SD	49.0 ± 24.2	47.4 ± 22.2	59.5 ± 20.3	50.5 ± 23.2	36.4 ± 28.2	68.0 ± 20.7	57.9 ± 19.4	67.9 ± 17.5	78.4 ± 21.6	74.1 ± 19.4	66.7 ± 23.8	21.1 ± 5.2	62.9 ± 22.6	78.4 ± 22.8	22.5 ± 4.0	36.2 ± 4.3
Age																	
	p	0.734	0.563	**0.032**	0.290	0.447	0.354	0.885	0.186	0.647	0.093	**0.016**	0.467	0.723	0.095	0.217	0.654
≤ 57 years	Mean ± SD	52.1 ± 23.2	47.1 ± 20.3	61.3 ± 18.7	53.2 ± 22.6	35.9 ± 26.8	69.9 ± 19.3	58.3 ± 19.3	66.4 ± 16.3	79.5 ± 20.3	75.3 ± 18.8	73.2 ± 22.5	21.7 ± 4.9	62.8 ± 21.0	79.3 ± 21.6	23.2 ± 3.7	36.3 ± 4.1
> 57 years	Mean ± SD	50.3 ± 22.2	49.3 ± 21.5	56.7 ± 19.7	52.1 ± 23.2	38.7 ± 28.1	71.8 ± 21.8	59.5 ± 21.1	69.3 ± 19.6	77.8 ± 22.0	70.5 ± 19.3	65.4 ± 24.1	21.1 ± 5.2	64.8 ± 23.5	74.0 ± 23.6	22.7 ± 2.7	36.5 ± 4.3
Interval to onset																	
	**p**	**0.011**	**0.001**	**0.026**	0.081	**0.005**	**0.028**	0.069	**0.044**	0.975	0.967	0.419	**0.003**	**0.010**	0.742	0.034	0.703
≤ 55 months	Mean ± SD	56.0 ± 24.6	53.1 ± 23.5	62.1 ± 21.2	55.6 ± 24.6	42.3 ± 30.1	74.7 ± 20.3	61.3 ± 21.5	70.8 ± 18.6	79.6 ± 20.5	73.5 ± 20.9	70.9 ± 25.9	22.4 ± 5.1	68.2 ± 23.8	77.2 ± 22.2	23.5 ± 3.6	36.6 ± 3.9
> 55 months	Mean ± SD	46.1 ± 19.6	42.9 ± 16.9	56.5 ± 17.0	48.6 ± 21.0	29.5 ± 22.9	67.0 ± 20.4	56.2 ± 18.4	65.3 ± 17.6	79.6 ± 21.5	74.6 ± 17.3	69.4 ± 20.4	20.3 ± 5.0	59.1 ± 21.0	78.4 ± 21.8	22.4 ± 3.6	36.9 ± 3.3
Idiopathic																	
	p	**0.011**	**0.001**	** < 0.001**	**0.009**	** < 0.001**	**0.007**	**0.008**	**0.016**	0.307	0.443	**0.011**	**0.001**	** > 0.001**	0.131	**0.002**	**0.002**
Yes	Mean ± SD	56.9 ± 24.9	55.0 ± 23.8	64.6 ± 19.3	58.1 ± 23.6	45.2 ± 29.9	75.6 ± 21.3	63.4 ± 20.1	71.9 ± 17.7	80.4 ± 21.4	74.5 ± 19.7	75.3 ± 24.4	22.8 ± 5.0	72.6 ± 23.2	79.4 ± 22.1	23.7 ± 3.9	37.1 ± 4.4
No	Mean ± SD	47.8 ± 21.0	44.0 ± 17.6	55.1 ± 18.3	48.5 ± 21.0	31.3 ± 24.2	68.2 ± 19.8	56.4 ± 19.7	65.9 ± 17.7	78.2 ± 20.6	72.9 ± 18.4	65.5 ± 21.5	20.3 ± 5.0	57.9 ± 19.4	75.0 ± 22.4	22.2 ± 3.5	36.0 ± 3.9
Inflammatory																	
	p	0.181	0.758	0.063	0.479	0.695	0.973	0.375	0.783	0.997	0.985	0.475	0.916	0.762	0.654	0.459	0.471
Yes	Mean ± SD	47.8 ± 23.0	47.5 ± 19.7	54.7 ± 21.3	51.2 ± 21.4	35.6 ± 26.7	71.2 ± 20.4	57.6 ± 20.7	68.0 ± 18.4	79.9 ± 19.4	74.1 ± 17.8	72.4 ± 19.4	21.7 ± 4.2	62.7 ± 19.4	76.9 ± 19.3	22.6 ± 3.5	36.5 ± 3.1
No	Mean ± SD	53.1 ± 23.2	49.3 ± 21.7	60.6 ± 18.5	53.3 ± 23.0	38.1 ± 28.0	71.6 ± 20.9	60.1 ± 20.0	68.7 ± 17.9	79.0 ± 21.4	73.5 ± 19.3	69.2 ± 24.3	21.3 ± 5.3	64.9 ± 23.2	77.0 ± 23.2	23.0 ± 3.8	36.5 ± 4.4
Neoplastic																	
	p	0.052	**0.002**	**0.012**	**0.036**	**0.008**	** < 0.001**	0.100	**0.036**	0.062	0.500	** < 0.001**	** < 0.001**	** < 0.001**	0.367	0.061	**0.016**
Yes	Mean ± SD	43.5 ± 18.6	38.8 ± 11.5	51.0 ± 14.7	42.7 ± 19.3	24.7 ± 18.9	59.8 ± 17.1	54.5 ± 19.9	62.5 ± 16.4	72.9 ± 21.4	71.9 ± 18.6	55.6 ± 21.7	18.0 ± 5.0	48.8 ± 18.9	73.3 ± 23.9	21.6 ± 3.9	34.7 ± 5.1
No	Mean ± SD	53.3 ± 23.6	50.5 ± 22.0	60.7 ± 19.6	54.6 ± 22.8	39.7 ± 28.4	73.5 ± 20.7	60.4 ± 20.1	69.6 ± 18.0	80.3 ± 20.8	73.9 ± 19.1	72.3 ± 22.7	22.0 ± 4.9	67.1 ± 21.9	77.6 ± 22.1	23.1 ± 3.7	36.8 ± 3.9
Postoperative																	
	p	0.772	0.160	0.581	0.677	0.137	0.914	0.267	0.607	0.681	0.599	**0.048**	0.122	0.104	0.360	0.233	0.399
Yes	Mean ± SD	54.3 ± 20.5	42.0 ± 16.4	57.6 ± 16.3	50.8 ± 22.2	29.2 ± 23.3	72.3 ± 15.8	55.0 ± 15.6	66.3 ± 14.2	80.4 ± 22.1	71.5 ± 18.6	61.4 ± 18.7	19.8 ± 5.7	57.1 ± 18.0	71.8 ± 26.1	22.2 ± 3.4	36.2 ± 3.7
No	Mean ± SD	51.6 ± 23.6	49.8 ± 21.7	59.5 ± 19.7	53.1 ± 22.8	38.6 ± 28.1	71.4 ± 21.3	60.1 ± 20.7	68.9 ± 18.4	79.0 ± 20.9	73.9 ± 19.0	71.0 ± 23.6	21.6 ± 5.0	65.4 ± 22.8	77.7 ± 21.8	23.0 ± 3.8	36.5 ± 4.2
Therapy*																	
	**p**	0.253	0.328	0.630	0.316	0.542	0.393	0.309	0.983	0.254	0.108	0.988	0.648	0.516	0.740	0.523	0.694
Yes	Mean ± SD	51.2 ± 22.8	48.4 ± 20.9	59.5 ± 19.5	52.4 ± 22.1	37.1 ± 27.2	71.9 ± 20.4	59.1 ± 20.2	68.7 ± 18.0	79.6 ± 21.0	74.6 ± 17.8	70.1 ± 22.8	21.3 ± 5.1	64.2 ± 21.5	77.2 ± 22.0	22.8 ± 3.7	36.4 ± 4.2
No	Mean ± SD	58.4 ± 26.4	53.1 ± 24.8	57.2 ± 17.1	57.6 ± 27.3	41.9 ± 32.3	67.6 ± 23.7	63.6 ± 19.9	67.4 ± 17.4	75.2 ± 21.1	64.7 ± 26.8	68.5 ± 28.2	21.8 ± 5.4	66.5 ± 30.8	74.6 ± 25.7	23.3 ± 4.3	36.8 ± 3.7
Drug-treated																	
	p	0.121	**0.003**	**0.004**	**0.020**	**0.005**	**0.003**	**0.017**	**0.017**	0.239	0.538	**0.021**	**0.011**	**0.002**	0.320	0.273	0.625
Yes	Mean ± SD	54.1 ± 24.3	53.0 ± 23.0	63.0 ± 21.2	56.7 ± 23.7	42.9 ± 29.0	75.6 ± 19.8	62.6 ± 19.3	71.4 ± 17.4	80.9 ± 19.8	74.6 ± 18.1	73.4 ± 23.1	22.2 ± 5.0	69.4 ± 22.4	78.7 ± 21.4	23.1 ± 3.9	36.4 ± 4.5
No	Mean ± SD	49.5 ± 21.7	44.4 ± 18.3	55.2 ± 16.1	48.7 ± 20.9	31.7 ± 25.1	67.1 ± 20.9	56.2 ± 20.6	65.5 ± 18.1	77.3 ± 22.1	72.6 ± 19.9	66.1 ± 23.0	20.5 ± 5.1	59.0 ± 21.3	75.1 ± 23.2	22.7 ± 3.5	36.5 ± 2.6
Training																	
	p	**0.025**	0.057	0.472	0.427	0.060	0.214	0.082	0.059	0.162	0.777	0.063	**0.024**	**0.029**	0.138	**0.011**	0.280
Yes	Mean ± SD	48.2 ± 21.4	46.7 ± 20.0	58.8 ± 19.1	52.0 ± 21.1	34.4 ± 26.1	69.9 ± 20.0	57.3 ± 20.1	66.7 ± 17.1	77.3 ± 22.1	74.3 ± 17.8	67.5 ± 22.8	20.8 ± 4.9	61.7 ± 21.0	75.0 ± 23.0	22.3 ± 3.8	36.4 ± 4.1
No	Mean ± SD	56.9 ± 24.6	51.8 ± 22.6	60.0 ± 19.6	54.1 ± 24.7	41.8 ± 29.4	73.7 ± 21.7	62.6 ± 19.9	71.2 ± 18.8	81.9 ± 19.1	72.7 ± 20.6	73.3 ± 23.6	22.2 ± 5.3	68.2 ± 23.8	79.8 ± 21.1	23.7 ± 3.5	36.6 ± 4.1
Physical therapy																	
	p	** < 0.001**	** < 0.001**	**0.012**	**0.001**	** < 0.001**	** < 0.001**	**0.004**	**0.036**	**0.038**	0.899	**0.001**	** < 0.001**	** < 0.001**	**0.020**	**0.019**	**0.005**
Yes	Mean ± SD	45.9 ± 20.0	43.0 ± 16.1	56.1 ± 17.2	48.2 ± 19.5	30.8 ± 22.5	67.1 ± 19.4	56.1 ± 20.5	66.5 ± 18.6	76.7 ± 21.5	73.9 ± 17.3	65.6 ± 21.7	20.1 ± 4.9	57.5 ± 17.5	74.6 ± 21.6	22.4 ± 3.7	36.1 ± 3.9
No	Mean ± SD	60.5 ± 4.8	57.3 ± 24.7	64.0 ± 21.2	59.7 ± 25.2	47.3 ± 31.5	77.8 ± 21.1	64.5 ± 18.7	71.5 ± 16.7	82.8 ± 19.7	73.3 ± 20.7	76.2 ± 24.2	23.3 ± 4.8	74.7 ± 24.9	80.5 ± 23.0	23.6 ± 3.7	37.1 ± 4.4
Surgery																	
	p	0.052	** < 0.001**	**0.001**	**0.002**	** < 0.001**	** < 0.001**	**0.001**	**0.002**	**0.003**	0.122	** < 0.001**	**0.001**	** < 0.001**	**0.002**	**0.002**	**0.010**
Yes	Mean ± SD	47.4 ± 20.0	40.6 ± 16.5	53.6 ± 16.3	46.0 ± 20.5	26.6 ± 23.0	64.7 ± 18.6	53.4 ± 20.5	63.1 ± 16.9	73.5 ± 22.7	71.3 ± 18.2	60.3 ± 21.2	19.8 ± 5.3	54.6 ± 19.3	70.7 ± 23.6	21.9 ± 3.5	35.7 ± 4.3
No	Mean ± SD	54.5 ± 24.6	53.6 ± 22.2	62.6 ± 20.1	56.8 ± 23.0	43.9 ± 28.3	75.5 ± 21.0	61.3 ± 19.2	71.8 ± 17.9	82.5 ± 19.2	75.0 ± 19.3	75.4 ± 22.7	22.3 ± 4.8	70.2 ± 22.2	80.6 ± 20.8	23.5 ± 3.8	36.9 ± 4.0

*SD* standard deviation, FACE-Q paralysis module, *AE* adverse event.

*Any therapy.

Significant values are in bold.

### Multivariate analysis of independent associations between clinical parameters between FACE-Q subdomains

Tables 3, 4 and 5 present the results of the multivariate analysis. Three models were calculated, with the first model (Table 3) including univariate significant clinical parameters, the second model (Table 4) including physical therapy and the third model (Table 5) including surgery in addition. Significant associations between clinical parameters and FACE-Q domains were found in the linear regression models. The interval between facial palsy onset and the survey as well as idiopathic cause were significantly associated with the subdomain *Appearance Face*, with a longer interval having a negative effect and an idiopathic cause having a positive effect on the results. For each additional month that the patient was affected by facial palsy, the Rasch score decreased by 8.6 points (95% CI 2.09–15.18; p = 0.010), i.e. the longer the onset was, the better was the facial function based on the FACE-Q domains. Patients with idiopathic cause scored 8.79 points higher (95% CI 2.19–15.37; p = 0.009), i.e. better facial function, than patients with known etiology. Patients with a shorter duration (< 55 months) scored higher compared to patients with a longer duration (> 55 months) in six domains: *Appearance Eyes, Appearance Face, Appearance Smile, Appearance Distress, Eye Function, Facial Function*. An idiopathic cause of facial nerve palsy was significantly associated with higher scores in all domains except the subdomains *Speech*, *Breathing* and *Speech Function*. Gender was independently related to three domains, including *Appearance Lips*, *Appearance Distress*, and *Eating/Drinking*. In these domains, males obtained higher scores compared to females. Age was only associated with the domain *Eating/Drinking*, with patients aged 57 and younger achieving higher scores than older patients.

**Table 3:** Multivariate model 1: independent associations between clinical parameters and the domains of the FACE-Q.

Domains	Regression coefficient B	95% CI		Standard error	p
		Lower limit	Upper limit		
Appearance eyes					
Sex (0 = male; 1 = female)	− 6.156	− 12.938	0.625	3.436	0.075
Interval/onset (0 = ≤ 55; 1 ≥ 55 months)	− 8.622	− 15.168	− 2.076	3.317	0.010
Idiopathic (0 = no; 1 = yes)	8.780	2.191	15.368	3.338	0.009
Appearance face					
Sex (0 = male; 1 = female)	− 2.822	− 8.942	3.297	3.101	0.364
Interval to onset (0 = ≤ 55 months; 1 ≥ 55 months)	− 8.986	− 14.902	− 3.070	2.998	0.003
Idiopathic (0 = no; 1 = yes)	9.987	4.026	15.948	3.020	0.001
Appearance forehead					
Age (0 = ≤ 57 years; 1 ≥ 57 years)	− 4.540	− 10.232	1.152	2.883	0.117
Interval/onset (0 = ≤ 55; 1 ≥ 55 months	− 4.635	− 10.362	1.092	2.901	0.112
Idiopathic (0 = no; 1 = yes)	9.801	4.022	15.579	2.927	0.001
Appearance lips					
Sex (0 = male; 1 = female)	− 6.571	− 12.714	− 0.428	3.116	0.036
Idiopathic (0 = no; 1 = yes)	9.929	3.911	15.948	3.053	0.001
Appearance smile					
Interval to onset (0 = ≤ 55 months; 1 ≥ 55 months)	− 11.189	− 18.909	− 3.468	3.912	0.005
Idiopathic (0 = no; 1 = yes)	13.950	6.168	21.732	3.943	0.001
Appearance distress					
Sex (0 = male; 1 = female)	− 9.199	− 15.219	− 3.178	3.051	0.003
Interval/onset (0 = ≤ 55; 1 ≥ 55 months	− 6.389	− 12.222	− 0.557	2.955	0.032
Idiopathic (0 = no; 1 = yes)	7.469	1.583	13.355	2.982	0.013
Psychological function					
Sex (0 = male; 1 = female)	− 4.565	− 10.083	0.953	2.799	0.104
Idiopathic (0 = no; 1 = yes)	7.220	1.811	12.630	2.744	0.009
Social function					
Interval/onset (0 = ≤ 55; 1 ≥ 55 months	− 4.656	− 9.948	0.636	2.681	0.084
Idiopathic (0 = no; 1 = yes)	7.134	1.800	12.468	2.703	0.009
Eating/drinking					
Sex (0 = male; 1 = female)	− 7.838	− 14.316	− 1.361	3.284	0.018
Age (0 = ≤ 57 years; 1 ≥ 57 years)	− 7.497	− 13.834	− 1.160	3.213	0.021
Idiopathic (0 = no; 1 = yes)	10.489	4.088	16.889	3.245	0.001
Eye function					
Interval/onset (0 = ≤ 55; 1 ≥ 55 months	− 1.823	− 3.276	− 0.369	0.737	0.014
Idiopathic (0 = no; 1 = yes)	2.602	1.136	4.067	0.743	0.001
Facial function					
Interval/onset (0 = ≤ 55; 1 ≥ 55 months	− 7.242	− 13.555	− 0.929	3.199	0.025
Idiopathic (0 = no; 1 = yes)	15.130	8.765	21.495	3.225	< 0.001
Eye adverse effect					
Interval/onset (0 = ≤ 55; 1 ≥ 55 months	− 0.919	− 1.962	0.124	0.528	0.084
Idiopathic (0 = no; 1 = yes)	1.711	0.659	2.763	0.533	0.002

Significant parameters in univariate tests were included.

*CI* confidence interval.

**Table 4:** Multivariate model 2: Independent associations between clinical parameters including also physical therapy and the domains of the FACE-Q.

Domains	Regression coefficient B	95% CI		Standard error	p
		Lower limit	Upper limit		
Appearance eyes					
Sex (0 = male; 1 = female)	− 5.594	− 12.241	1.053	3.368	0.099
Interval/onset (0 = ≤ 55; 1 ≥ 55 months)	− 6.515	− 13.075	0.045	3.323	0.052
Idiopathic (0 = no; 1 = yes)	7.455	0.947	13.963	3.297	0.025
Physical therapy (0 = no;1 = yes)	− 10.064	− 16.811	− 3.316	3.419	0.004
Appearance face					
Sex (0 = male; 1 = female)	− 2.502	− 8.468	3.464	3.023	0.409
Interval/onset (0 = ≤ 55; 1 ≥ 55 months)	− 6.704	− 12.636	− 0.773	3.005	0.027
Idiopathic (0 = no; 1 = yes)	8.524	2.647	14.402	2.978	0.005
Physical therapy (0 = no;1 = yes)	− 9.981	− 16.105	− 3.857	3.103	0.002
Appearance forehead					
Age (0 = ≤ 57 years; 1 ≥ 57 years)	− 4.841	− 10.444	0.762	2.838	0.090
Interval/onset (0 = ≤ 55; 1 ≥ 55 months)	− 2.991	− 8.761	2.779	2.923	0.308
Idiopathic (0 = no; 1 = yes)	8.663	2.914	14.412	2.912	0.003
Physical therapy (0 = no;1 = yes)	− 7.810	− 13.769	− 1.851	3.018	0.011
Appearance lips					
Sex (0 = male; 1 = female)	− 5.606	− 11.666	0.454	3.074	0.070
Idiopathic (0 = no; 1 = yes)	8.034	1.999	14.068	3.061	0.009
Physical therapy (0 = no;1 = yes)	− 9.358	− 15.483	− 3.234	3.107	0.003
Appearance smile					
Interval/onset (0 = ≤ 55; 1 ≥ 55 months)	− 9.034	− 16.856	− 1.212	3.963	0.024
Idiopathic (0 = no; 1 = yes)	12.620	4.863	20.376	3.930	0.002
Physical therapy (0 = no;1 = yes)	− 9.790	− 17.840	− 1.741	4.079	0.017
Appearance distress					
Sex (0 = male; 1 = female)	− 8.817	− 14.726	− 2.907	2.994	0.004
Interval/onset (0 = ≤ 55; 1 ≥ 55 months)	− 4.475	− 10.346	1.396	2.975	0.134
Idiopathic (0 = no; 1 = yes)	6.245	0.411	12.078	2.956	0.036
Physical therapy (0 = no;1 = yes)	− 8.706	− 14.754	− 2.658	3.065	0.005
Psychological function					
Sex (0 = male; 1 = female)	− 3.904	− 9.382	1.575	2.779	0.162
Idiopathic (0 = no; 1 = yes)	5.819	0.356	11.282	2.771	0.037
Physical Therapy (0 = no;1 = yes)	− 6.885	− 12.416	− 1.353	2.806	0.015
Social function					
Interval/onset (0 = ≤ 55; 1 ≥ 55 months)	− 3.691	− 9.103	1.721	2.742	0.180
Idiopathic (0 = no; 1 = yes)	6.539	1.172	11.906	2.719	0.017
Physical therapy (0 = no;1 = yes)	− 4.382	− 9.951	1.187	2.822	0.122
Eating/drinking					
Sex (0 = male; 1 = female)	− 7.375	− 13.803	− 0.947	3.259	0.025
Age (0 = ≤ 57 years; 1 ≥ 57 years)	− 7.853	− 14.137	− 1.570	3.186	0.015
Idiopathic (0 = no; 1 = yes)	9.117	2.660	15.573	3.273	0.006
Physical therapy (0 = no;1 = yes)	− 7.276	− 13.809	− 0.743	3.312	0.029
Eye function					
Interval/onset (0 = ≤ 55; 1 ≥ 55 months)	− 1.314	− 2.770	0.142	0.738	0.077
Idiopathic (0 = no; 1 = yes)	2.261	0.814	3.709	0.733	0.002
Physical therapy (0 = no;1 = yes)	− 2.359	− 3.863	− 0.855	0.762	0.002
Facial function					
Interval/onset (0 = ≤ 55; 1 ≥ 55 months)	− 4.463	− 10.684	1.758	3.152	0.159
Idiopathic (0 = no; 1 = yes)	13.271	7.087	19.456	3.134	< 0.001
Physical therapy (0 = no;1 = yes)	− 12.894	− 19.318	− 6.471	3.255	< 0.001
Eye adverse effect					
Interval/onset (0 = ≤ 55; 1 ≥ 55 months)	− 0.758	− 1.827	0.312	0.542	0.164
Idiopathic (0 = no; 1 = yes)	1.611	0.550	2.672	0.538	0.003
Physical therapy (0 = no;1 = yes)	− 0.725	− 1.827	0.377	0.558	0.196
Face adverse effect					
Idiopathic (0 = no; 1 = yes)	0.930	− 0.202	2.062	0.574	0.107
Physical therapy (0 = no;1 = yes)	− 0.829	− 1.974	0.316	0.581	0.155

Significant parameters in univariate tests were included.

*CI* confidence interval.

**Table 5:** Multivariate model 3: independent associations between clinical parameters including also surgery and the domains of the FACE-Q.

Domains	Regression coefficient B	95% CI		Standard error	p
		Lower limit	Upper limit		
Appearance face					
Sex (0 = male; 1 = female)	− 3.728	− 9.812	2.357	3.083	0.228
Interval/onset (0 = ≤ 55; 1 ≥ 55 months)	− 8.214	− 14.086	− 2.342	2.975	0.006
Idiopathic (0 = no; 1 = yes)	6.677	0.195	13.160	3.284	0.044
Surgery (0 = no; 1 = yes)	− 8.134	− 14.829	− 1.439	3.392	0.018
Appearance forehead					
Age (0 = ≤ 57 years; 1 ≥ 57 years)	− 4.105	− 9.809	1.599	2.889	0.157
Interval/onset (0 = ≤ 55; 1 ≥ 55 months)	− 4.249	− 9.981	1.483	2.903	0.145
Idiopathic (0 = no; 1 = yes)	7.781	1.405	14.156	3.229	0.017
Surgery (0 = no; 1 = yes)	− 4.804	− 11.308	1.700	3.294	0.147
Appearance lips					
Sex (0 = male; 1 = female)	− 7.155	− 13.243	− 1.066	3.088	0.021
Idiopathic (0 = no; 1 = yes)	6.160	− 0.513	12.833	3.385	0.070
Surgery (0 = no; 1 = yes)	− 8.589	− 15.487	− 1.692	3.499	0.015
Appearance smile					
Interval/onset (0 = ≤ 55; 1 ≥ 55 months)	− 10.248	− 17.937	− 2.558	3.896	0.009
Idiopathic (0 = no; 1 = yes)	9.903	1.361	18.446	4.328	0.023
Surgery (0 = no; 1 = yes)	− 9.583	− 18.329	− 0.837	4.432	0.032
Appearance distress					
Sex (0 = male; 1 = female)	− 10.299	− 16.251	− 4.347	3.016	0.001
Interval/onset (0 = ≤ 55; 1 ≥ 55 months)	− 5.457	− 11.212	0.298	2.916	0.063
Idiopathic (0 = no; 1 = yes)	3.594	− 2.773	9.960	3.226	0.267
Surgery (0 = no; 1 = yes)	− 9.469	− 16.036	− 2.902	3.327	0.005
Psychological function					
Sex (0 = male; 1 = female)	− 5.216	− 10.669	0.237	2.766	0.061
Idiopathic (0 = no; 1 = yes)	3.553	− 2.388	9.493	3.013	0.240
Surgery (0 = no; 1 = yes)	− 8.565	− 14.707	− 2.423	3.116	0.007
Social function					
Interval/onset (0 = ≤ 55; 1 ≥ 55 months)	− 3.931	− 9.183	1.322	2.661	0.141
Idiopathic (0 = no; 1 = yes)	4.016	− 1.818	9.850	2.956	0.176
Surgery (0 = no; 1 = yes)	− 7.384	− 13.358	− 1.411	3.027	0.016
Eating/drinking					
Sex (0 = male; 1 = female)	− 8.831	− 15.158	− 2.503	3.208	0.006
Age (0 = ≤ 57 years; 1 ≥ 57 years)	− 6.163	− 12.375	0.050	3.150	0.052
Idiopathic (0 = no; 1 = yes)	4.764	− 2.271	11.798	3.566	0.183
Surgery (0 = no; 1 = yes)	− 12.634	− 19.855	− 5.412	3.661	0.001
Eye function					
Interval/onset (0 = ≤ 55; 1 ≥ 55 months)	− 1.702	− 3.155	− 0.249	0.736	0.022
Idiopathic (0 = no; 1 = yes)	2.004	0.388	3.621	0.819	0.015
Surgery (0 = no; 1 = yes)	− 1.412	− 3.063	0.239	0.837	0.093
Facial function					
Interval/onset (0 = ≤ 55; 1 ≥ 55 months)	− 6.454	− 12.699	− 0.210	3.164	0.043
Idiopathic (0 = no; 1 = yes)	11.227	4.280	18.175	3.520	0.002
Surgery (0 = no; 1 = yes)	− 9.230	− 16.325	− 2.134	3.595	0.011
Eye adverse effect					
Interval/onset (0 = ≤ 55; 1 ≥ 55 months)	− 0.841	− 1.886	0.205	0.530	0.114
Idiopathic (0 = no; 1 = yes)	1.355	0.195	2.515	0.588	0.022
Surgery (0 = no; 1 = yes)	− 0.853	− 2.039	0.334	0.601	0.158
Face adverse effect					
Idiopathic (0 = no; 1 = yes)	0.677	− 0.563	1.918	0.629	0.283
Surgery (0 = no; 1 = yes)	− 0.954	− 2.234	0.325	0.649	0.143

Significant parameters in univariate tests were included.

*CI* confidence interval.

Additionally, physical therapy was included in in another multivariate model (Table 4). It emerged as an independent predictor for the subdomains of *Appearance* (*Eyes, Face, Forehead, Lips, Smile*), as well as *Appearance Distress, Psychological Function, Eating/Drinking* and *Eye Function*. Patients who participated in physical therapy showed lower scores, indicating more impairment, than those who did not undergo physical therapy. The strongest effect was seen in the subdomain *Facial* Function, where patients who participated in physical therapy scored 12.89 points lower (95% CI 6.47–19.32; p < 0.001) than those who did not. Even taking these effects into account, the parameter idiopathic cause remained an independent predictor for all mentioned FACE-Q domains. Similarly, when the parameter surgery was added in a third model (Table 5), it was found to be independently and significantly associated with subdomains *Appearance Face, Appearance Eyes, Appearance Smile, Appearance Distress, Psychological Function, Social Function, Eating/Drinking* and *Facial Function*. Patients who did not undergo surgery displayed higher scores than those who received surgery. The strongest effect was seen in the subdomain *Eating/Drinking*, where patients who underwent surgery scored 12.63 points lower (95% CI 5.41–19.86; p = 0.001) than those who did not.

### Factor analysis

Supplementary Table 6 shows the normality tests for the questionnaire scales. The KMO = 0.889 confirmed the suitability for factor analysis. Bartlett’s test of sphericity, x^2^ = 2645.98 (p < 0.001), showed significantly high correlations between items for MLFA. Three factors in combination were able to explain 65.72% of the variance. The scree plot justified keeping three factors. Supplementary Table 7 shows the factor loadings after rotation. Based on the given original structure of the FACE-Q (main domains), the items that cluster on the same factor suggest that factor 1 represents appearance, factor 2 function and factor 3 quality of life. We performed the same analysis twice, once including all items and once excluding the two checklists (*Eye Adverse Effect, Face Adverse Effect*). Both analyses gave very similar results (KMO = 0.897; x^2^ = 2389.57; p < 0.001) with the importance that three factors were found.

## Discussion

Patient-reported outcome (PROM) instruments can provide valuable information about patients’ subjective quality of life. Patients with facial palsy often suffer from the disease for several months or even life-long, and their overall quality of life is impaired^20^. The results of the PROMs can be used clinically with these patients to tailor therapy to their individual needs and improve their quality of life. To ensure reliable use, an instrument must be validated and its reliability demonstrated in the target language and cultural context^13^.

The present study showed that the translated German version of FACE-Q Paralysis has good to excellent consistency, as described for the original English version^8^. No difficulties were encountered in the translation process due to cultural differences. Patients had no difficulty in clearly understanding individual questions in German. The German version showed good to excellent internal validity, except for the subdomain *Breathing*. Cronbach’s alpha ranged from 0.77 to 0.97. For the original English version, the values ranged from 0.78 to 0.96. In both this study and the original version, the subdomain *Breathing* had a lower alpha of 0.61 and 0.71, respectively^8^. Thus, the values for the German version were in a similar range as those of the original English version. A possible reason for the lower internal consistency for this subdomain could be that it assesses many different aspects related to breathing, such as breathing while eating, sleeping or exercising. Thus, the construct measured may be highly diverse, resulting in a lower internal consistency^21^. The correlations within the FACE-Q Paralysis show higher intercorrelations within the scales of each domain than with other domains, as was also shown for the original English version^8^.

A major aim of this study was to investigate the domain *Appearance*, which is not covered by other validated PROMs such as the FDI and the FaCE^5,15,16^. On average, the domain *Appearance* consistently produced lower scores across all subdomains. The mean scores within the domain *Appearance* ranged from 37.5 to 59.3, while the mean scores of the other domains ranged from 59.5 to 79.9. The subdomain *Smile* had the lowest mean score of 37.5. According to previous research, the visibility of the teeth and the position of the upper lip are crucial predictive variables of attractiveness^22^. In patients with facial palsy, these aspects are often affected, as they are unable to achieve a meaningful excursion when smiling, even with maximum effort^23^. The patient’s perception of this altered smile can be confirmed by the outcome scores of the FACE-Q domain *Smile*. IN contrast, the subdomain *Forehead* had the highest score of 59.3. The forehead is known to have a lower correlation with overall attractiveness than other facial features^24^. It is often less visibly affected by motor impairments and can be easily covered by hairstyles, hats or other accessories. While the appearance of the forehead may remain relatively unchanged, there may be limitations in the ability to furrow or raise the eyebrows. In general, the lower the score, the greater the impairment and the higher the level of distress about one’s appearance. Dissatisfaction with one’s appearance may contribute to lower self-esteem. This has already been shown in a previous study by Norris et al.^25^. It is important to clinically assess this aspect of self-perception at an early stage in order to offer psychological support to patients if needed.

To determine which parameters might predict FACE-Q Paralysis scores, we further investigated the influence of potentially contributing factors on the scores. Indeed, the following factors were found to be independent negative predictors for the PROM: longer interval to the onset of the palsy (> 55 months), female sex, age (> 57 years), physical therapy and surgery. On the other hand, an idiopathic cause of facial paralysis correlated positively with FACE-Q scores. In contrast, other etiologies and other adjuvant therapies did not significantly predict scores. In the domain *Appearance*, the presence of an idiopathic cause of facial palsy was unexpectedly a significant predictor in all subdomains, correlating with higher scores on the FACE-Q. The subdomains *Appearance Distress, Psychological Function, Social Function, Eating/Drinking, Eye Function, Facial Function* and *Eye Adverse Effect* were also positively influenced by the presence of an idiopathic cause. The median interval form the survey to the onset of the palsy of patients with idiopathic cause was 4 years. Given that idiopathic paresis has the best prognosis and therefore the highest likelihood of recovery, it may well be that many of the patients with idiopathic causes were already within the range of probable recovery and therefore suffered less disability^26^. In addition, patients with idiopathic facial palsy are more often affected by paresis than by paralysis. This could also lead to higher scores for this group^27^. Other etiologies were not found to be significant in this study.

Longer interval to the onset of the palsy (> 55 months) correlated with lower scores in the subdomains *Appearance Eye, Face and Smile*, as well as *Appearance Distress*, *Eye Function* and *Facial Function*. As all recruited patients presented to the Facial Nerve Centre, it can be assumed that patients with long-term problems, who are not affected by rapid recovery, were more likely to be included in this study^28^. Another reason for lower scores in the domain *Appearance* could be the shift in focus due to a longer interval to the onset of the paralysis. The focus on facial functions, which is mainly present in the acute phase, may be compensated by a longer interval or by a habituation effect, and be replaced by a focus on limitations in appearance^28^.

As expected from previous studies, longer interval to onset of the palsy was not shown to be a significant predictor in the subdomains *Psychological* and *Social Function*, among others^29^. These aspects of quality of life may have adapted over time^30^. Satisfaction with appearance, on the other hand, which is negatively affected by longer interval, may remain unchanged or even worsen over time.

The two variables physical therapy and surgery turned out to be independent predictors in several subdomains*.* Patients who had undergone physical therapy or surgery had lower scores, indicating greater impairment in these subdomains. Rather obviously, patients who are in need of such therapies tend to have greater severity of facial palsy. Reconstruction surgery is mainly performed in patients with facial paralysis, with much more severe facial dysfunction. This is reflected in lower scores. It would be interesting to carry out further research and compare the results of the FACE-Q before and after respective treatment to determine possible changes in quality of life due to the treatment.

The main limitations of the study are, on the one hand, the selection of the parameters considered as possible predictors and, on the other hand, the presence of selection bias. Since all included patients were recruited through a facial nerve center, it can be assumed that more severe cases, more chronic than acute cases and patients specifically seeking therapy to improve their impairments were included in this study than would be expected in a more representative sample of individuals affected by facial palsy^28^. A more comprehensive examination should be carried out to identify further possible factors influencing the questionnaire, such as comorbidity and current status of palsy. We further cannot exclude selection bias due to the effect that 73% of the patients contacted did not answer at least one of the questionnaires. In addition, no objective assessment of facial nerve function was recorded. In a future survey, this could even be provided by the patients themselves, for example using the Sunnybrook grading, to investigate the relationship between subjective perception and a functional assessment^31^.

## Conclusion

The German version of the FACE-Q paralysis module works well in adult patients with facial nerve palsy. We were able to identify predictors in our cohort for the different scales. Knowledge of these influencing factors can be useful for clinicians in order to reduce the psychological impact of facial nerve palsy and provide early supportive interventions in areas of individual importance to patients.

### Supplementary Information

Supplementary Tables.

## Data Availability

The data presented in this study are available on request from the corresponding author.
