# Supplementary material for: Translation and validation of the German version of the FACE-Q paralysis module in adult patients with unilateral peripheral facial palsy
Source: Sci Rep. 2024 Mar 31;14:7606. doi: 10.1038/s41598-024-58159-8 (PMC10982296; doi:10.1038/s41598-024-58159-8)
Supplement: Supplementary file 1 — Supplementary Tables. [file 41598_2024_58159_MOESM1_ESM.docx]

**Title Page**

**Translation and Validation of the German version of the FACE-Q Paralysis Module in Adult Patients with Unilateral Peripheral Facial Palsy**

Wieta Elin Moritz^1^, Gerd Fabian Volk^1,2,3^, Helene Kreysa^4^, Orlando Guntinas-Lichius^1,2,3^

**7 Supplement Tables**

**Supplement Tables**

**Supplement Table 1**

| **Supplement Table 1. Internal consistency of the FACE-Q, FDI and FaCE scales** | | | |
| --- | --- | --- | --- |
| **Questionnaire** | **Cronbach‘s α** | **95%-CI** | |
| **FACE-Q** |  | Lower bound | Upper bound |
| **Appearance** |  |  |  |
| Eyes (N=211) | 0.954 | 0.944 | 0.963 |
| Face (N=212) | 0.945 | 0.933 | 0.955 |
| Forehead (N=212) | 0.941 | 0.928 | 0.952 |
| Lips (N=212) | 0.950 | 0.939 | 0.960 |
| Smile (N=213) | 0.972 | 0.966 | 0.978 |
| **Health-related Quality of Life** |  |  |  |
| Appear Distress (N=213) | 0.920 | 0.903 | 0.936 |
| Psychological (N=213) | 0.950 | 0.939 | 0.959 |
| Social Function (N=213) | 0.924 | 0.908 | 0.939 |
| Speech Distress (N=212) | 0.919 | 0.902 | 0.935 |
| **Function** |  |  |  |
| Breathing (N=214) | 0.609 | 0.523 | 0.684 |
| Eating/Drinking (N=214) | 0.906 | 0.886 | 0.924 |
| Eyes (N=213) | 0.828 | 0.790 | 0.861 |
| Face (N=212) | 0.890 | 0.867 | 0.911 |
| Speech (N=214) | 0.948 | 0.937 | 0.957 |
| **Adverse Effects** |  |  |  |
| Eyes (N=214) | 0.771 | 0.721 | 0.815 |
| Face (N=214) | 0.832 | 0.797 | 0.864 |
| **FDI** |  |  |  |
| Physical Function (N=213) | 0.857 | 0.825 | 0.886 |
| Social / Well-being Function (N=212) | 0.791 | 0.743 | 0.833 |
| **FaCE** |  |  |  |
| Facial Movement (N=209) |  |  |  |
| Facial Comfort(N=213) | 0.828 | 0.783 | 0.865 |
| Oral Function (N=214) | 0.893 | 0.865 | 0.916 |
| Eye Comfort (N=214) | 0.828 | 0.775 | 0.869 |
| Lacrimal Control (N=213) | 0.759 | 0.684 | 0.816 |
| Social Function (N=213) | N/A |  |  |
| Total Score (N=209) | 0.868 | 0.836 | 0.895 |

SD = standard deviation; CI = confidence interval; N/A = not available, because this domain consists of only one question

| **Supplement Table 2. Correlation between FACE-Q and FDI and FaCE*** | | | | | | | | | |
| --- | --- | --- | --- | --- | --- | --- | --- | --- | --- |
|  | **FDI** | | **FaCE** | | | | | | |
|  | Physical Function | Social / Well-being Function | Facial Movement | Facial Comfort | Oral Function | Eye Comfort | Lacrimal Function | Social Function | Total Score |
|  | **rho** | **rho** | **rho** | **rho** | **rho** | **rho** | **rho** | **rho** | **rho** |
| **FACE-Q** |  |  |  |  |  |  |  |  |  |
| **Appearance** |  |  |  |  |  |  |  |  |  |
| Eyes (N=211) | 0.467 | 0.369 | 0.473 | 0.533 | 0.407 | 0.333 | 0.280 | 0.510 | 0.598 |
| Face (N=212) | 0.547 | 0.385 | 0.677 | 0.568 | 0.516 | 0.431 | 0.330 | 0.608 | 0.718 |
| Forehead (N=212) | 0.509 | 0.425 | 0.481 | 0.527 | 0.486 | 0.375 | 0.333 | 0.483 | 0.600 |
| Lips (N=212) | 0.488 | 0.316 | 0.496 | 0.538 | 0.469 | 0.307 | 0.333 | 0.478 | 0.590 |
| Smile (N=213) | 0.533 | 0.394 | 0.715 | 0.518 | 0.548 | 0.373 | 0.370 | 0.562 | 0.699 |
| **Health-related Quality of Life** |  |  |  |  |  |  |  |  |  |
| Appear Distress (N=213) | 0.533 | 0.478 | 0.547 | 0.517 | 0.523 | 0.427 | 0.286 | 0.748 | 0.724 |
| Psychological (N=213) | 0.429 | 0.546 | 0.409 | 0.480 | 0.367 | 0.313 | 0.225 | 0.611 | 0.576 |
| Social Function (N=213) | 0.457 | 0.655 | 0.381 | 0.464 | 0.438 | 0.292 | 0.235 | 0.679 | 0.594 |
| Speech Distress (N=212) | 0.545 | 0.434 | 0.380 | 0.426 | 0.528 | 0.307 | 0.276 | 0.639 | 0.587 |
| **Function** |  |  |  |  |  |  |  |  |  |
| Breathing (N=214) | 0.474 | 0.405 | 0.203 | 0.290 | 0.411 | 0.329 | 0.287 | 0.362 | 0.398 |
| Eating/Drinking (N=214) | 0.758 | 0.409 | 0.531 | 0.542 | 0.787 | 0.500 | 0.418 | 0.562 | 0.725 |
| Eyes (N=213) | 0.704 | 0.366 | 0.640 | 0.563 | 0.606 | 0.678 | 0.429 | 0.570 | 0.773 |
| Face (N=212) | 0.743 | 0.422 | 0.786 | 0.616 | 0.739 | 0.532 | 0.395 | 0.630 | 0.828 |
| Speech (N=214) | 0.635 | 0.392 | 0.442 | 0.409 | 0.579 | 0.391 | 0.268 | 0.525 | 0.576 |
| **Adverse Effects** |  |  |  |  |  |  |  |  |  |
| Eyes (N=214) | 0.669 | 0.407 | 0.442 | 0.538 | 0.556 | 0.758 | 0.572 | 0.492 | 0.712 |
| Face (N=214) | 0.561 | 0.372 | 0.375 | 0.735 | 0.515 | 0.387 | 0.291 | 0.518 | 0.640 |

**Supplement Table 2**

*All values were statistically significant at p<0.001; FACE-Q Paralysis Module; FDI = Facial Disability Index; FaCE = Facial Clinimetric Evaluation; Spearman’s rho

| **Supplement Table 3. Correlations within FACE-Q subdomains** | | | | | | | | | | | | |
| --- | --- | --- | --- | --- | --- | --- | --- | --- | --- | --- | --- | --- |
|  | **FACE-Q** | | | | | | | | | | | |
|  | Eyes | | Face | | Forehead | | Lips | | Smile | | Appearance Distress | |
|  | **rho** | **p** | **rho** | **p** | **rho** | **p** | **rho** | **p** | **rho** | **p** | **rho** | **p** |
| **FACE-Q** |  |  |  |  |  |  |  |  |  |  |  |  |
| **Appearance** |  |  |  |  |  |  |  |  |  |  |  |  |
| Eyes (N=211) | 1.000 | < 0.001 | 0.778 | < 0.001 | 0.602 | < 0.001 | 0.616 | < 0.001 | 0.583 | < 0.001 | 0.589 | < 0.001 |
| Face (N=212) | 0.778 | < 0.001 | 1.000 | < 0.001 | 0.662 | < 0.001 | 0.707 | < 0.001 | 0.836 | < 0.001 | 0.682 | < 0.001 |
| Forehead (N=212) | 0.602 | < 0.001 | 0.662 | < 0.001 | 1.000 | < 0.001 | 0.611 | < 0.001 | 0.560 | < 0.001 | 0.460 | < 0.001 |
| Lips (N=212) | 0.616 | < 0.001 | 0.707 | < 0.001 | 0.611 | < 0.001 | 1.000 | < 0.001 | 0.690 | < 0.001 | 0.509 | < 0.001 |
| Smile (N=213) | 0.583 | < 0.001 | 0.836 | < 0.001 | 0.560 | < 0.001 | 0.690 | < 0.001 | 1.000 | < 0.001 | 0.644 | < 0.001 |
| **Health-related Quality of Life** |  |  |  |  |  |  |  |  |  |  |  |  |
| Appear Distress (N=213) | 0.589 | < 0.001 | 0.682 | < 0.001 | 0.460 | < 0.001 | 0.509 | < 0.001 | 0.644 | < 0.001 | 1.000 | < 0.001 |
| Psychological (N=213) | 0.539 | < 0.001 | 0.604 | < 0.001 | 0.496 | < 0.001 | 0.491 | < 0.001 | 0.529 | < 0.001 | 0.693 | < 0.001 |
| Social Function (N=213) | 0.461 | < 0.001 | 0.529 | < 0.001 | 0.448 | < 0.001 | 0.366 | < 0.001 | 0.471 | < 0.001 | 0.677 | < 0.001 |
| Speech Distress (N=212) | 0.343 | < 0.001 | 0.454 | < 0.001 | 0.386 | < 0.001 | 0.385 | < 0.001 | 0.439 | < 0.001 | 0.580 | < 0.001 |
| **Function** |  |  |  |  |  |  |  |  |  |  |  |  |
| Breathing (N=214) | 0.150 | 0.030 | 0.260 | < 0.001 | 0.304 | < 0.001 | 0.203 | 0.003 | 0.206 | 0.002 | 0.320 | < 0.001 |
| Eating/Drinking (N=214) | 0.411 | < 0.001 | 0.531 | < 0.001 | 0.470 | < 0.001 | 0.480 | < 0.001 | 0.524 | < 0.001 | 0.560 | < 0.001 |
| Eyes (N=213) | 0.535 | < 0.001 | 0.620 | < 0.001 | 0.518 | < 0.001 | 0.440 | < 0.001 | 0.552 | < 0.001 | 0.589 | < 0.001 |
| Face (N=212) | 0.485 | < 0.001 | 0.654 | < 0.001 | 0.533 | < 0.001 | 0.525 | < 0.001 | 0.714 | < 0.001 | 0.649 | < 0.001 |
| Speech (N=214) | 0.239 | < 0.001 | 0.355 | < 0.001 | 0.396 | < 0.001 | 0.344 | < 0.001 | 0.366 | < 0.001 | 0.459 | < 0.001 |
| **Adverse Effects** |  |  |  |  |  |  |  |  |  |  |  |  |
| Eyes (N=214) | 0.448 | < 0.001 | 0.490 | < 0.001 | 0.416 | < 0.001 | 0.361 | < 0.001 | 0.448 | < 0.001 | 0.470 | < 0.001 |
| Face (N=214) | 0.401 | < 0.001 | 0.460 | < 0.001 | 0.388 | < 0.001 | 0.463 | < 0.001 | 0.383 | < 0.001 | 0.415 | < 0.001 |

**Supplement Table 3**

FACE-Q Paralysis Module; FDI = Facial Disability Index; FaCE = Facial Clinimetric Evaluation; Spearman’s rho

**Supplement Table 4**

| **Supplement Table 4. Correlations within FACE-Q subdomains** | | | | | | | | | | |
| --- | --- | --- | --- | --- | --- | --- | --- | --- | --- | --- |
|  | **FACE-Q** | | | | | | | | | |
|  | Psychological Function | | Social Function | | Speech Distress | | Breathing | | Eating and Drinking | |
|  | **rho** | **p** | **rho** | **p** | **rho** | **p** | **rho** | **p** | **rho** | **p** |
| **FACE-Q** |  |  |  |  |  |  |  |  |  |  |
| **Appearance** |  |  |  |  |  |  |  |  |  |  |
| Eyes (N=211) | 0.539 | < 0.001 | 0.461 | < 0.001 | 0.343 | < 0.001 | 0.150 | 0.030 | 0.411 | < 0.001 |
| Face (N=212) | 0.604 | < 0.001 | 0.529 | < 0.001 | 0.454 | < 0.001 | 0.260 | < 0.001 | 0.531 | < 0.001 |
| Forehead (N=212) | 0.496 | < 0.001 | 0.448 | < 0.001 | 0.386 | < 0.001 | 0.304 | < 0.001 | 0.470 | < 0.001 |
| Lips (N=212) | 0.491 | < 0.001 | 0.366 | < 0.001 | 0.385 | < 0.001 | 0.203 | 0.003 | 0.480 | < 0.001 |
| Smile (N=213) | 0.529 | < 0.001 | 0.471 | < 0.001 | 0.439 | < 0.001 | 0.206 | 0.003 | 0.524 | < 0.001 |
| **Health-related Quality of Life** |  |  |  |  |  |  |  |  |  |  |
| Appear Distress (N=213) | 0.693 | < 0.001 | 0.677 | < 0.001 | 0.580 | < 0.001 | 0.320 | < 0.001 | 0.560 | < 0.001 |
| Psychological (N=213) | 1.000 | < 0.001 | 0.798 | < 0.001 | 0.491 | < 0.001 | 0.308 | < 0.001 | 0.385 | < 0.001 |
| Social Function (N=213) | 0.798 | < 0.001 | 1.000 | < 0.001 | 0.607 | < 0.001 | 0.387 | < 0.001 | 0.461 | < 0.001 |
| Speech Distress (N=212) | 0.491 | < 0.001 | 0.607 | < 0.001 | 1.000 | < 0.001 | 0.430 | < 0.001 | 0.565 | < 0.001 |
| **Function** |  |  |  |  |  |  |  |  |  |  |
| Breathing (N=214) | 0.308 | < 0.001 | 0.387 | < 0.001 | 0.430 | < 0.001 | 1.000 | < 0.001 | 0.437 | < 0.001 |
| Eating/Drinking (N=214) | 0.385 | < 0.001 | 0.461 | < 0.001 | 0.565 | < 0.001 | 0.437 | < 0.001 | 1.000 | < 0.001 |
| Eyes (N=213) | 0.435 | < 0.001 | 0.446 | < 0.001 | 0.503 | < 0.001 | 0.324 | < 0.001 | 0.599 | < 0.001 |
| Face (N=212) | 0.442 | < 0.001 | 0.459 | < 0.001 | 0.538 | < 0.001 | 0.303 | < 0.001 | 0.720 | < 0.001 |
| Speech (N=214) | 0.377 | < 0.001 | 0.477 | < 0.001 | 0.732 | < 0.001 | 0.516 | < 0.001 | 0.589 | < 0.001 |
| **Adverse Effects** |  |  |  |  |  |  |  |  |  |  |
| Eyes (N=214) | 0.395 | < 0.001 | 0.396 | < 0.001 | 0.388 | < 0.001 | 0.432 | < 0.001 | 0.518 | < 0.001 |
| Face (N=214) | 0.357 | < 0.001 | 0.361 | < 0.001 | 0.416 | < 0.001 | 0.237 | < 0.001 | 0.492 | < 0.001 |

FACE-Q Paralysis Module; FDI = Facial Disability Index; FaCE = Facial Clinimetric Evaluation; Spearman’s rho

**Supplement Table 5**

| **Supplement Table 5. Correlations within FACE-Q subdomains** | | | | | | | | | | |
| --- | --- | --- | --- | --- | --- | --- | --- | --- | --- | --- |
|  | **FACE-Q** | | | | | | | | | |
|  | Eye Function | | Facial Function | | Speech Function | | Eye Adverse Effect | | Face Adverse Effect | |
|  | **rho** | **p** | **rho** | **p** | **rho** | **p** | **rho** | **p** | **rho** | **p** |
| **FACE-Q** |  |  |  |  |  |  |  |  |  |  |
| **Appearance** |  |  |  |  |  |  |  |  |  |  |
| Eyes (N=211) | 0.535 | < 0.001 | 0.485 | < 0.001 | 0.239 | < 0.001 | 0.448 | < 0.001 | 0.401 | < 0.001 |
| Face (N=212) | 0.620 | < 0.001 | 0.654 | < 0.001 | 0.355 | < 0.001 | 0.490 | < 0.001 | 0.460 | < 0.001 |
| Forehead (N=212) | 0.518 | < 0.001 | 0.533 | < 0.001 | 0.396 | < 0.001 | 0.416 | < 0.001 | 0.388 | < 0.001 |
| Lips (N=212) | 0.440 | < 0.001 | 0.525 | < 0.001 | 0.344 | < 0.001 | 0.361 | < 0.001 | 0.463 | < 0.001 |
| Smile (N=213) | 0.552 | < 0.001 | 0.714 | < 0.001 | 0.366 | < 0.001 | 0.448 | < 0.001 | 0.383 | < 0.001 |
| **Health-related Quality of Life** |  |  |  |  |  |  |  |  |  |  |
| Appear Distress (N=213) | 0.589 | < 0.001 | 0.649 | < 0.001 | 0.459 | < 0.001 | 0.470 | < 0.001 | 0.415 | < 0.001 |
| Psychological (N=213) | 0.435 | < 0.001 | 0.442 | < 0.001 | 0.377 | < 0.001 | 0.395 | < 0.001 | 0.357 | < 0.001 |
| Social Function (N=213) | 0.446 | < 0.001 | 0.459 | < 0.001 | 0.477 | < 0.001 | 0.396 | < 0.001 | 0.361 | < 0.001 |
| Speech Distress (N=212) | 0.503 | < 0.001 | 0.538 | < 0.001 | 0.732 | < 0.001 | 0.388 | < 0.001 | 0.416 | < 0.001 |
| **Function** |  |  |  |  |  |  |  |  |  |  |
| Breathing (N=214) | 0.324 | < 0.001 | 0.303 | < 0.001 | 0.516 | < 0.001 | 0.432 | < 0.001 | 0.237 | < 0.001 |
| Eating/Drinking (N=214) | 0.599 | < 0.001 | 0.720 | < 0.001 | 0.589 | < 0.001 | 0.518 | < 0.001 | 0.492 | < 0.001 |
| Eyes (N=213) | 1.000 | < 0.001 | 0.768 | < 0.001 | 0.541 | < 0.001 | 0.707 | < 0.001 | 0.482 | < 0.001 |
| Face (N=212) | 0.768 | < 0.001 | 1.000 | < 0.001 | 0.579 | < 0.001 | 0.568 | < 0.001 | 0.513 | < 0.001 |
| Speech (N=214) | 0.541 | < 0.001 | 0.579 | < 0.001 | 1.000 | < 0.001 | 0.433 | < 0.001 | 0.405 | < 0.001 |
| **Adverse Effects** |  |  |  |  |  |  |  |  |  |  |
| Eyes (N=214) | 0.707 | < 0.001 | 0.568 | < 0.001 | 0.433 | < 0.001 | 1.000 | < 0.001 | 0.489 | < 0.001 |
| Face (N=214) | 0.482 | < 0.001 | 0.513 | < 0.001 | 0.405 | < 0.001 | 0.489 | < 0.001 | 1.000 | < 0.001 |

FACE-Q Paralysis Module; Spearman’s rho

**Supplement Table 6**

| **Supplement Table 6. Normality test of FACE-Q, FDI and FaCE scales** | | | | | | |
| --- | --- | --- | --- | --- | --- | --- |
| **Questionnaire** | **Skewness** | **SE** | **Kurtosis** | **SE** | **Shapiro-Wilk-Test** | |
|  |  |  |  |  | **W** | **p** |
| **FACE-Q** |  |  |  |  |  |  |
| **Appearance** |  |  |  |  |  |  |
| Eyes (N=211) | 0.371 | 0.172 | -0.047 | 0.343 | 0.958 | <0.001 |
| Face (N=212) | 0.710 | 0.172 | 0.475 | 0.343 | 0.943 | <0.001 |
| Forehead (N=212) | 0.468 | 0.172 | 0.173 | 0.343 | 0.944 | <0.001 |
| Lips (N=212) | 0.433 | 0.172 | 0.032 | 0.343 | 0.949 | <0.001 |
| Smile (N=213) | 0.527 | 0.172 | -0.410 | 0.343 | 0.945 | <0.001 |
| **Health-related Quality of Life** |  |  |  |  |  |  |
| Appear Distress (N=213) | -0.166 | 0.172 | -0.838 | 0.343 | 0.938 | <0.001 |
| Psychological (N=213) | -0.112 | 0.172 | 0.355 | 0.343 | 0.974 | <0.001 |
| Social Function (N=213) | 0.157 | 0.172 | -0.419 | 0.343 | 0.953 | <0.001 |
| Speech Distress (N=212) | -1.013 | 0.172 | 0.945 | 0.343 | 0.860 | <0.001 |
| **Function** |  |  |  |  |  |  |
| Breathing (N=214) | -0.417 | 0.172 | -0.598 | 0.343 | 0.948 | <0.001 |
| Eating/Drinking (N=214) | -0.215 | 0.172 | -1.124 | 0.343 | 0.925 | <0.001 |
| Eyes (N=213) | 0.474 | 0.172 | -0.895 | 0.343 | 0.929 | <0.001 |
| Face (N=212) | .0214 | 0.172 | -0.747 | 0.343 | 0.928 | <0.001 |
| Speech (N=214) | -0.807 | 0.172 | 0.108 | 0.343 | 0.878 | <0.001 |
| **Adverse Effects** |  |  |  |  |  |  |
| Eyes (N=214) | -0.535 | 0.172 | -0.311 | 0.343 | 0.948 | <0.001 |
| Face (N=214) | -2.386 | 0.172 | 9.618 | 0.343 | 0.773 | <0.001 |
| **FDI** |  |  |  |  |  |  |
| Physical Function (N=213) | -0.495 | 0.172 | -0.673 | 0.343 | 0.938 | <0.001 |
| Social / Well-being Function (N=212) | -0.681 | 0.172 | -0.219 | 0.343 | 0.944 | <0.001 |
| **FaCE** |  |  |  |  |  |  |
| Facial Movement (N=209) | 0.290 | 0.172 | -1.196 | 0.343 | 0.915 | <0.001 |
| Facial Comfort(N=213) | -0.434 | 0.172 | -0.982 | 0.343 | 0.917 | <0.001 |
| Oral Function (N=214) | -1.379 | 0.172 | 1.411 | 0.343 | 0.803 | <0.001 |
| Eye Comfort (N=214) | -0.309 | 0.172 | -1.331 | 0.343 | 0.888 | <0.001 |
| Lacrimal Control (N=213) | -0.810 | 0.172 | -0.428 | 0.343 | 0.833 | <0.001 |
| Social Function (N=213) | -1.278 | 0.172 | 0.644 | 0.343 | 0.780 | <0.001 |
| Total Score (N=209) | -0.318 | 0.172 | -0.567 | 0.343 | 0.965 | <0.001 |

SE = standard error; W = test value Shapiro-Wilk-Test

**Supplement Table 7**

| **Supplement Table 7. Rotated exploratory factor analysis results for FACE-Q questionnaire scales** | | | |
| --- | --- | --- | --- |
|  | **Varimax rotated factor loadings** | | |
|  | **Factor 1**  Appearance | **Factor 2**  Function | **Factor 3**  Quality of Life |
| **FACE-Q** |  |  |  |
| **Appearance** |  |  |  |
| Eyes (N=211) | 0.767 |  |  |
| Face (N=212) | 0.889 |  |  |
| Forehead (N=212) | 0.671 |  |  |
| Lips (N=212) | 0.737 |  |  |
| Smile (N=213) | 0.835 |  |  |
| **Health-related Quality of Life** |  |  |  |
| Appear Distress (N=213) | 0.499 | 0.439 | 0.472 |
| Psychological (N=213) | 0.420 |  | 0.757 |
| Social Function (N=213) | 0.314 | 0.347 | 0.804 |
| Speech Distress (N=212) |  | 0.721 | 0.413 |
| **Function** |  |  |  |
| Breathing (N=214) |  | 0.535 |  |
| Eating/Drinking (N=214) | 0.372 | 0.699 |  |
| Eyes (N=213) | 0.493 | 0.623 |  |
| Face (N=212) | 0.604 | 0.664 |  |
| Speech (N=214) |  | 0.821 |  |
| **Adverse Effects** |  |  |  |
| Eyes (N=214) | 0.376 | 0.544 |  |
| Face (N=214) |  | 0.522 |  |
